# Performance of LED Fluorescence Microscopy for the Diagnosis of Pulmonary Tuberculosis in HIV Positive Individuals in Addis Ababa, Ethiopia

**DOI:** 10.1155/2015/794064

**Published:** 2015-11-24

**Authors:** Konjit Getachew, Tamrat Abebe, Abebaw Kebede, Adane Mihret, Getachew Melkamu

**Affiliations:** ^1^Biomedical Institute, College of Health Science, Mekelle University, P.O. Box 1871, Mekelle, Ethiopia; ^2^Department of Microbiology, Immunology and Parasitology, School of Medicine, College of Health Science, Addis Ababa University, P.O. Box 9086, Addis Ababa, Ethiopia; ^3^Ethiopian Public Health Institute, P.O. Box 1248, Addis Ababa, Ethiopia; ^4^Armauer Hansen Research Institute, Addis Ababa, Ethiopia

## Abstract

*Background*. Despite its lower sensitivity, smear microscopy remains the main diagnostic method for pulmonary tuberculosis (PTB) in resource-limited countries as TB culturing methods like LJ (Lowenstein-Jensen) are expensive to use as a routine base. This study aimed to evaluate the performance of LED-FM for the diagnosis of PTB in HIV positive individuals.* Methods*. Cross-sectional study was conducted in Zewditu Memorial Hospital and Teklehaimanot Health Center HIV/ART clinics in Addis Ababa, Ethiopia. Each sample was stained with ZN and Auramine O staining and examined with bright-field microscope and LED-FM microscope, respectively. LJ culture was used as a reference.* Results*. Out of 178 study participants, twenty-four (13.5%) patients were confirmed as positive for MTB with LJ culture. The yield of ZN microscopy and LED-FM in direct and concentrated sample was 3.9%, 8.4%, 6.2%, and 8.4%, respectively. Sensitivity, specificity, positive predictive value (PPV), and negative predictive value (NPV) of direct ZN microscopy were 29.2%, 100%, 100%, and 90.1%, respectively, and of LED-FM microscopy in direct sputum sample were 62.5%, 100%, 100%, and 94.5%, respectively.* Conclusion*. LED-FM has better sensitivity for the diagnosis of PTB in HIV positive individuals as compared to conventional ZN microscopy. LED-FM can be used as an alternative to conventional ZN microscopy.

## 1. Introduction

Tuberculosis (TB) is an infectious disease caused by* Mycobacterium tuberculosis* (MTB). According to WHO 2013 report, there were an estimated 8.6 million incident cases of TB globally. From these, sub-Saharan Africa accounts for about 27% of the cases. The estimated number of TB cases in Ethiopia in the same year was about 230 per 100,000 populations [1]. The Acquired Immunodeficiency Syndrome (AIDS) epidemic has accelerated TB epidemic worldwide particularly in sub-Saharan Africa [2]. The risk of developing TB in HIV patients has increased at about 20–30 times compared with those who do not have HIV infection [3]. Survival rate of HIV positive individuals coinfected with TB is significantly reduced compared to those HIV monoinfected individuals. In addition, in the coinfected groups, tuberculosis is one of the major AIDS defining illnesses [4]. With an estimated 1.1 million people living with HIV, Ethiopia has one of the largest populations of HIV infected people in the world. However, HIV prevalence among the adult population is lower than many sub-Saharan African countries. Adult HIV prevalence in 2009 has been estimated to be between 1.4% and 2.8% [5].

Direct ZN sputum microscopy is the most widely used diagnostic method for pulmonary TB diagnosis in resource-limited settings. ZN microscopy is highly specific but the sensitivity varies from 20 to 80% [3, 6]. The current approach for pulmonary TB diagnosis in Ethiopia is mainly dependent on sputum microscopy with ZN staining with the sputum collected over two consecutive days spot-morning spot [5].

The diagnosis of pulmonary tuberculosis is more challenging in the HIV infected population as a result of immune dysregulation leading to more frequent paucibacillary disease, which has a direct impact on the performance of microscopy [7, 8]. Light Emitting Diode (LED) technology has been developed to allow benefit of fluorescent microscope without the associated cost. WHO recommended the use of LED microscope as an alternative to conventional fluorescent microscope in resource-limited settings [3]. LEDs provide a cheap and reliable light source with a more robust and long lifespan (>50,000 hours); additionally, no darkroom is required for their operation; LED microscopy has been shown to have equivalent specificity and improved sensitivity over conventional ZN microscopy [9]. Therefore, evaluation of LED fluorescence microscopy in diagnosing pulmonary tuberculosis in resource-limited countries like Ethiopia with a high burden of HIV and TB coinfection is likely to change management and contribute significantly to disease treatment and transmission.

## 2. Materials and Methods

### 2.1. Study Population

All HIV positive individuals who came to ART clinics of Zewditu Memorial Hospital and Teklehaimanot Health Center between December 2011 and June 2012 and fulfill the inclusion criteria were eligible for the study. Zewditu Memorial Hospital and Teklehaimanot Health Centers are found in Addis Ababa, Ethiopia. Both institutes are governmental institutions and give service to the public and have general inpatient and outpatient services.

### 2.2. Inclusion Criteria

We included children (>10 years old) and adults (>18 years old) in compliance with the sign and symptoms of TB using WHO standard and patients who are not on anti-TB treatment, who are willing to participate, and who provided informed consent. For participants between 10 and 18 years of age, consent from their guardians and assent from the study participant were mandatory for inclusion in the study. Patients on anti-TB treatment and HIV positive children less than 10 years of age were excluded from the study as this age group may not be able to produce a good quality sputum. Patients were enrolled to the study irrespective of their ART status. Patients who were not able to produce sputum were excluded from the study. Most of the study population is comprised mostly of clinic patients.

### 2.3. Ethical Clearance

The study protocol was reviewed and approved by the institutional review board of College of Health Science at Addis Ababa University and permission letter was obtained from Zewditu Memorial Hospital, Teklehaimanot Health Center, and EHNRI to conduct the study.

### 2.4. Patient Evaluation and Sample Collection

HIV positive patients who attended the ART clinics of the study site were screened for sign and symptoms of TB by the attending physicians using nonprobable convenient sampling method. After instructions on how to produce good quality sputum, specimens were received. Patients were asked to provide three (two spot and one early morning) sputum samples as per the national guideline over two consecutive days in a sterile, leak-proof, and screw-capped sputum cups which are labeled with patient code in the laboratory. The 1st sample was collected on the spot at initial consultation, the 2nd early morning at their home, and the 3rd on the spot by the time the patient delivered the early morning specimen to the ART clinics.

### 2.5. Laboratory Diagnosis Method

The specimens were transported immediately to the National TB Reference Laboratory at Ethiopian Public Health Institution (EPHI) using cold chain box and processed on the day of collection or stored overnight at 2–8°C and processed on the next day. In this study, we did two methods of smear preparation; one was prepared directly from the sputum and the second was made from the sediment after decontamination. The sputum samples were decontaminated using 4% NaOH (modified Petroff's method) and concentrated by centrifugation at 3000 ×g for 15 minutes after addition of phosphate buffer (pH 6.8). The concentrated specimen was used for inoculation onto the slant of Lowenstein-Jensen media for the early morning specimen only. We were not able to culture all the three samples due to resource limitation and we preferred to inoculate the early morning samples to increase the probability of detecting the true positives. Four smears were made per specimen (two from direct sputum and two from the concentrated sample). Each of the three samples from a single patient was stained with standard ZN staining method (Clin-Tech Ltd., Unit G Perram Works, UK) from both concentrated and direct samples. ZN stained slides were examined by bright-field microscopy (magnification ×1,000) on CX21 Olympus microscope. The number of acid fast bacilli (AFB) per 100 microscopic fields was reported as per WHO guidelines. On the other hand, for Auramine O staining, the same sample preparation is used but the smears are stained with Auramine O stain and counterstained with potassium permanganate for 60 seconds. The slides were read using the same microscope, equipped with the LED FluoLED fluorescence illuminator (magnification ×400). The number of acid fast bacilli (AFB) read per standard length of 2 cm long was reported. A length corresponding to 100 fields under 1000 magnifications was estimated to be equivalent to 20 fields under 400 magnifications. Two experienced laboratory technologists read the smears and they were blinded to the result of each other.

Early morning sputum samples were used for culturing MTB on Lowenstein-Jensen (LJ) media at Ethiopian Public Health Institute (EPHI) and decontaminated as indicated above and followed by neutralization using phosphate buffer pH of 6.8. For culture, 0.1 mL was used to inoculate the LJ culture media and incubated at 37°C for 8 weeks. The cultures were inspected each week and reported based on WHO guideline. ZN microscopy was used to confirm the presence of acid fast bacilli and strain identification was done using Capilia TB identification test.

### 2.6. Outcome Definition

The primary outcome for our analysis was to establish the performance of LED fluorescence microscopy when compared with the gold standard of positive LJ culture. Secondary outcome was that concentrated samples performed better with Ziehl-Neelsen microscopy but this difference was nullified when the LED fluorescence microscopy technique was used.

### 2.7. Sample Size Determination

The sample size was determined based on the prevalence rate of TB in HIV positive individuals (15%) reported by WHO in 2011 for Ethiopia. Nonprobable convenient sampling method and 95% confidence interval are used to calculate the sample size. The calculated sample size was 196.

### 2.8. Statistical Analysis

Statistical analysis was made using data collected from questionnaire and laboratory. All data were entered and cleared using Microsoft Office Excel 2007 and transported to IBM Statistical Package Social Science (SPSS) program, version 20, for analysis. The sensitivity, specificity, and positive and negative predictive ratio were calculated for both ZN and LED-FM methods compared with LJ culture as gold standard. Results were considered as significant at *p* < 0.1 and detection agreement between the methods was measured using Kappa value result.

### 2.9. Quality Assurance

Quality assurance process is as follows:

1. ZN/Auramin O: known positive (1+) and known negative panel smears were used.
2. Sputum sample processing for TB culture: start and end controls (sterile phosphate buffer solution) were used in batch of sputum sample processed for excluding cross contamination.
3. LJ media:
  - sterility test: 5% of LJ media prepared in each batch were incubated at 37°C for 48–72 hours. If any growth per tube medium is observed, the whole batch would not be used for testing.
  - performance test: reference strains of* M. tuberculosis* and nontuberculosis mycobacteria (*M. gordonae*) were inoculated onto newly prepared LJ media and the performance was assessed for mycobacterium growth within 14 days of incubation at 37°C. If there is growth of reference strains, the LJ media support the growth of mycobacteria and the prepared media can be used.

## 3. Result

### 3.1. Study Population

**Figure 1:**
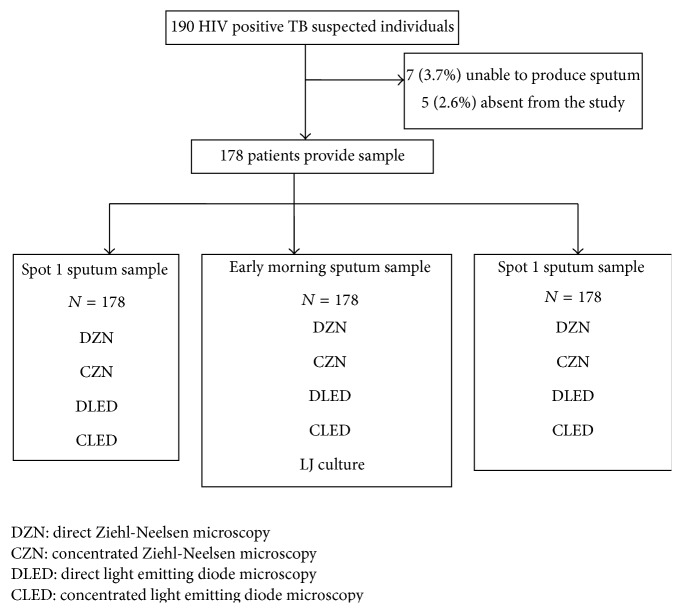
Study flow diagram.

From 190 HIV positive and eligible TB suspects, 178 (93.7%) provided 3 sputum samples and 12 of 190 were excluded from the study because they did not fulfill the inclusion criteria (Figure 1). Of the 178 HIV positive TB symptomatic patients, most of them were in the age group of 25–44 years (67.4%) with the median age of 37 ± 10.7 and the female to male ratio was 1.4 : 1 and in this population the culture proven TB prevalence was higher in males (62.5% versus 37.5%; AOR: 2.3; *p* < 0.1, CI 0.11–0.182) but there were limitations to this observation. The median for CD4+ T-lymphocyte was 324 cells/*μ*L (IQR 194.5–458) and despite not being statistically significant, patients with positive TB result had higher CD4+ T-lymphocyte than patients negative for TB (347 versus 321, *p* = 0.29), and 69.1% were on ART during the study (Table 1).

### 3.2. Microbiological Analysis

As shown in Table 2, five hundred and thirty-four (534) sputum samples were analyzed from 178 patients by ZN and LED-FM by direct and concentrated sample preparation. Five samples from the first spot sample, 7 from the early morning sample, and 2 from the second spot sample by ZN and 10 samples from spot 1 sample, 15 from early morning sample, and 5 from second spot sample by LED-FM were positive using direct sputum sample. Six samples from the first spot sample, 11 from the early morning sample, and 4 from the second spot sample were positive by CZN and 11 from the first spot sample, 15 from the early morning sample, and 5 from the second spot sample were positive by CLED-FM.

**Figure 2:**
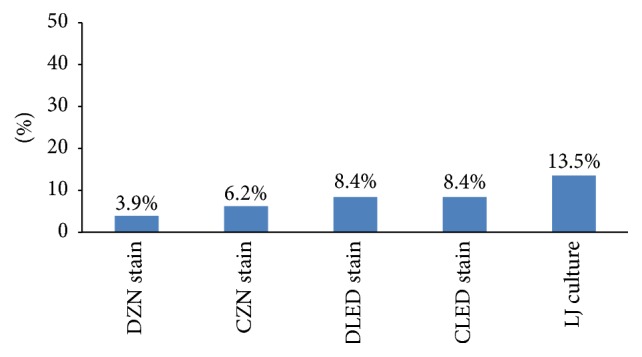
TB positivity of laboratory tests among TB suspected study participants.

Early morning sputum sample was used for culture and, of the 178 early morning sputum specimens, 24 (13.5%) were positive by LJ culture media (Figure 2). As LJ culture was considered as the reference standard for this study, LED-FM and ZN microscopy were compared and results for both ZN and LED-FM are presented for unconcentrated and concentrated sputum samples in Table 3. The values are calculated as per patient result. Positive microscopy is considered when at least one of the three consecutive samples is positive by smear microscopy as per WHO guideline for HIV positive individuals. Positive LJ culture is considered when bacterial colonies are seen within eight weeks of incubation. The culture contamination rate was zero. Among 24 culture positive MTB samples, the sensitivity, specificity, PPV, and NPV for DZN and CZN were 29.2%, 100%, 100%, and 90.1% and 48.8%, 100%, 100%, and 92.2%, respectively. The sensitivity, specificity, PPV, and NPV of DLED and CLED were the same with 62.5%, 100%, 100%, and 94.5%, respectively. The sensitivity of both DLED and CLED-FM was 62.5% (95% CI, 59.4% and 62.5%) and it was higher than DZN and CZN by 33.3% and 16.7%, respectively. The reading agreement between DZN and DLED-FM was *K* = 0.6 and CZN versus CLED-FM was *K* = 0.8. However, the specificity of LED-FM and ZN in both (unconcentrated and concentrated) sample preparations was similar (Table 3).

As shown in Figure 2, the diagnosis of TB has increased from 7 (3.9%) patients by DZN to 11 (6.2%) by CZN. The case detection rate further increased by both DLED-FM and CLED-FM to 15 (8.4%) and to 24 (13.5%) by LJ culture.

## 4. Discussion

In 2011, WHO recommended that conventional FM needs to be replaced by LED in all settings where conventional fluorescence microscope is used and that LED microscopy can be used as an alternative for conventional ZN microscopy in all levels of health laboratories [3]. In this study, the performance of LED-FM was evaluated in HIV positive pulmonary tuberculosis suspected patients and the sensitivity of LED was higher (62.5%) compared to ZN (29.2%) but equally specific (100%) in smears prepared from direct sputum samples.

Our finding is comparable with study conducted in Uganda (69.8%) [10] and South Africa (57%) [11]. However, it is low compared to another study done in South Africa that reports sensitivity of 84.7% [12] and in Kenya that reports sensitivity of 73.2% [9] and one study conducted in multicounty level including Ethiopia (77%) [13]. The lower sensitivity of our study finding could be explained by the fact that our study participants were limited to only HIV positive patients. This can have major impact on the smear positivity rate as well as the sensitivity of the test methods due to presence of small number of bacilli in the sputum in these groups.

In our study, the specificity of LED fluorescence microscopy in the direct sputum sample was 100%. This finding is comparable with another study done in Uganda (99.2%) [14] and two studies in South Africa which reported 98.9% [12] and 99% [11].

The sensitivity of ZN microscopy in this study was 29.2% in direct sputum samples. This low sensitivity of ZN microscopy was also comparable with study conducted in South Africa which reported 39% [11] in nonconcentrated sputum. However, our finding is lower than a study finding in Kenya that reported a sensitivity of 72% [9] and this could be due to the difference in the study participants as our study participants were HIV positive.

The sensitivity of the microscopic methods had been evaluated in smears prepared from the sediments of sputum after processing with 4% NaOH. The sensitivity of LED in both techniques (direct and concentrated) was not affected. A study conducted in Kenya comparing the sensitivity of LED in smear prepared from sediment reported a sensitivity of 78.5% [9], which is higher than our finding. This lower sensitivity in our study finding might be due to the difference in the reagents that we used for the concentration of the sputum. In our study, we used 4% NaOH but in the Kenyan study they used NaOCl. Additionally, in our study, the study participants were HIV positive but in their study they included study participants irrespective of their HIV status.

On the other hand, the sensitivity of ZN microscopy increased twice by using smears from the sediment. The agreement between the two methods (direct ZN and concentrated ZN) was good (*k*: 0.77). Additionally, the agreement between direct ZN and direct LED was also good (*k*: 0.62). In this study, the sensitivity and specificity of LED microscopy are unaffected by using direct or concentrated sputum samples.

In this study, we included study participants irrespective of the status of ART initiation. The study is unable to show the effect of ART initiation on the sensitivity and specificity of the test methods due to time and resource limitation. It would have been good if we would be able to study the performances separately. The sample size is also very small which may not be representative of the population. As liquid culture is more sensitive than solid medium culture, it would have been good if we have used it but for our study we used solid culture which may lead to underestimation of the culture positivity rate that would overestimate the performance of microscopic methods.

In conclusion, LED fluorescence microscopy has better performance for the diagnosis of PTB in HIV positive individuals compared to conventional ZN microscopy. Concentration technique improves the performance of ZN microscopy; however, it does not affect the performance of LED microscopy. Therefore, these findings support the use of LED fluorescence microscopy in HIV positive population as recommended by the WHO.

## Figures and Tables

**Table 1:** Characteristics of study population by tuberculosis culture status.

Characteristics	Total (*n* = 178)	Culture positive (*n* = 24)	Culture negative (*n* = 154)	OR	*p* value
Age, median (IQR)	37 (30–44)	35 (28–41)	34 (30–45)		0.57
Gender, male *n* (%)	73 (41)	15 (62.5)	58 (37.6)	AOR = 2.3	0.064 90% CI (0.011–0.182)
CD4 count, median (IQR)	324 (194.5–458)	347 (149–535)	321 (203–458)		0.29
Started ART *n* (%)	123 (69.1)	8 (33.3)	115 (74.7)		0.37
Clinical presentation *n* (%)					
Night sweating	125 (70.2)	20 (83.3)	105 (68.2)		0.13
Weight loss	112 (63)	17 (71)	95 (61.7)		0.38
Cough > 2 weeks	140 (78.6)	20 (83.3)	120 (78)		0.54

**Table 2:** Smear AFB detection and grading in spot-early morning-spot sputum specimens using direct and concentrated ZN and LED-FM microscopy method per sample analysis.

Grading	Methods											
	ZN *n* = 178						LED-FM *n* = 178					
	DZN *n* = 178			CZN *n* = 178			DLED-FM *n* = 187			CLED-FM *n* = 178		
Samples	S1 *n* (%)	EM *n* (%)	S2 *n* (%)	S1 *n* (%)	EM *n* (%)	S2 *n* (%)	S1 *n* (%)	EM *n* (%)	S2 *n* (%)	S1 *n* (%)	EM *n* (%)	S2 *n* (%)

Negative	173 (97.2)	171 (96.1)	176 (98.9)	172 (96.6)	167 (93.8)	174 (97.7)	168 (94.4)	163 (91.6)	173 (97.2)	167 (93.8)	163 (91.6)	173 (97.2)

Positive	5 (2.8)	7 (3.9)	—	6 (3.4)	11 (6.2)	4 (2.2)	10 (5.6)	15 (8.4)	5 (2.8)	11 (6.2)	15 (8.4)	5 (2.8)

**Table 3:** Performance of smear microscopy using Ziehl-Neelsen and LED-FM in TB suspected HIV positive individuals per patient analysis.

	ZN *n* = 24		Kappa value	LED-FM *n* = 24		Kappa value
	Direct	Concentrated		Direct	Concentrated	
Sensitivity % (90% CI)	29.2 (26.9–32.1)	45.8 (41.2–50.4)	0.767	62.5 (56.25–68.75)	62.5 (56.25–68.75)	0.743
Positive (%)	7 (3.9)	11 (6.2)		15 (8.4)	15 (8.4)	
Specificity % (90% CI)	100 (90–100)	100 (90–100)		100 (90–100)	100 (90–100)	
PPV %	100	100		100	100	
NPV %	90.1	92.2		94.5	94.5	
